# The ideal habitat for leaf-cutting ant queens to build their nests

**DOI:** 10.1038/s41598-022-08918-2

**Published:** 2022-03-22

**Authors:** Kátia K. A. Sousa, Roberto S. Camargo, Nadia Caldato, Adriano P. Farias, Marcus V. C. Calca, Alexandre Dal Pai, Carlos A. O. Matos, José C. Zanuncio, Isabel C. L. Santos, Luiz C. Forti

**Affiliations:** 1 Departamento de Proteção Vegetal, Faculdade de Ciências Agronômicas, Universidade Estadual Paulista (UNESP), Botucatu, São Paulo 18603-970 Brazil; 2 Departamento de Bioprocesso e Biotecnologia, Faculdade de Ciências Agronômicas, Universidade Estadual Paulista (UNESP), Botucatu, São Paulo 18603-970 Brazil; 3 Instituto de Ciências e Engenharia, Universidade Estadual Paulista, Itapeva, São Paulo 18409-010 Brazil; 4 Departamento de Entomologia/BIOAGRO, Universidade Federal de Viçosa, Viçosa, Minas Gerais 36570-900 Brazil; 5 Laboratório de Fitossanidade (FitLab), Instituto Federal de Mato Grosso, Cáceres, Mato Grosso 78201-380 Brazil

**Keywords:** Ecology, Evolution, Systems biology, Ecology

## Abstract

Queens of *Atta sexdens* Forel (Hymenoptera: Formicidae) face biotic and abiotic environmental factors in the environment while establishing their nests. Biotic factors such as predation, microbial pathogens, successful symbiotic fungus regurgitation, excavation effort and abiotic factors such as radiant sunlight, temperature, density, and soil moisture exert selection pressures on ant queens. Biotic factors such as temperature and solar irradiation affect the survival of the initial colony differently, in different environments in the field. Queens of the leaf-cutting ant *A. sexdens*, were installed in sunny and shaded conditions to test this hypothesis. Two hundred *A. sexdens* queens were collected and individualized in two experimental areas (sunny and shaded), each in an experimental area (25 m^2^) in the center of a square (50 × 50 cm). Temperature, irradiance, nest depth, rainfall and queen mortality were evaluated. *Atta sexdens* colony development was better in the shaded environment, and the depth and volume of the initial chamber, fungus garden biomass and number of eggs, larvae, pupae and workers were greater. The queen masses were similar in both environments but mortality was higher in the sunny environment. The worse parameter values for *A. sexdens* nests in the sunny environment are due to the greater solar irradiance, increasing the variation range of the internal temperature of the initial chamber of the nest. On the other hand, the more stable internal temperature of this chamber in the shaded environment, is due to the lower incidence of solar irradiance, which is also more advantageous for queen survival and the formation and development of *A. sexdens* colonies. Shaded environments are a better micro habitat for nesting *A. sexdens* than sunny ones.

## Introduction

The vulnerability of *Atta sexdens* Forel (Hymenoptera: Formicidae) queens is high during the claustral phase when they maximize nest excavation and protect themselves against environmental variables with minimal energy expenditure to increase their survival^1^. This phase starts after the “nuptial flight” when the queen excavates the first chamber of the nest, on average 10–30 cm deep, connected to the ground surface by a tunnel built and subsequently closed, which will be reopened when the first ant workers emerge^1^. The claustral queen loses about 40% of her weight by not eating and using her body reserves to cultivate the symbiotic fungus. In addition, it cares for and feeds its offspring with its body reserves, until the first adult workers start foraging, usually 3–4 months after the nuptial flight^2,3^.

The survival of the initial leaf-cutting ant colony varies according to biotic and abiotic environmental factors in the habitat where the queen establishes the nest. *Atta sexdens* queens prefer to found their nests in clearings^4^, possibly because they are easier and safer areas for landing without passing through the plant canopy^5^. In addition, the success of symbiotic fungus regurgitation, the minimization of excavation effort, and the more favorable soil temperature, density, and moisture may explain the greater queen success in these locations^1,2,6–9^.

Environmental and soil temperatures vary with solar irradiation and affect leaf-cutting ant nesting, as reported for *Atta* species in forests (shaded places) and pastures (sunny places)^10–12^. The initial depth of the chamber exerts the greatest selection pressure (selective forces), varying between ant species, with deeper chambers for *A. bisphaerica* than for *A. sexdens rubropilosa*^13^. The nesting process, habits, and foraging strategies differed between *A. bisphaerica* and *A. sexdens*, with the former normally establishing their nests in full sun in areas with predominantly grass forages and the latter in shaded areas where it cuts dicot leaves^14^. Furthermore, differences in nest depth among these species may be related to soil temperature, which is lower in shaded areas^7^. Fungal chambers in exposed nests in pastures are deeper than those in shaded areas within forests with soil temperature negatively correlated with depth^7^. Soil humidity and temperature act simultaneously due to the thermo preference of ant workers building shallow nests in cold soils and deeper nests in warm ones^7^. Humidity also varies with soil depth, affecting nest-digging behavior by leaf-cutting ants^7,15^.

Solar irradiation and, consequently, temperature, affected the depth of the initial chamber and the survival of leaf-cutting ant colonies. The survival of the founding *A. sexdens* queen and the development of the initial colony of this leaf-cutting ant, during the claustral phase, differ between sunny and shaded environments. As a consequence, survival of the founding *A. sexdens* queen and the development of their initial colony will be better in shaded environments. The objective was to evaluate the numbers of eggs, larvae, pupae, and adults, queen mass and fungus garden biomass and the dimensions (width, length, height, and depth) of the *A. sexdens* nests in soils in sunny and shaded environments. As a consequence, queen mortality during the claustral phase was also obtained.

## Results

**Figure 1:**
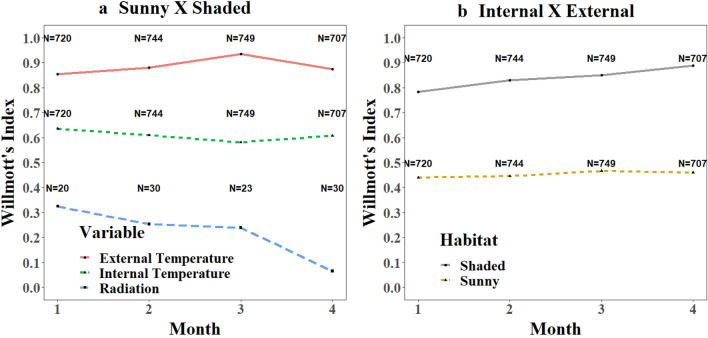
Temperature and irradiance over the first 4 months of *Atta sexdens* (Hymenoptera: Formicidae) nests in sunny and shaded environments. Willmott similarity index between irradiance and inside and outside temperatures (**a**) and between outside and inside temperatures (**b**) in sunny and shaded environments. (N = sample size).

The Willmott index indicates that over the first 4 months, the similarity (proximity) of the outdoor temperature data (month 1 = 0.85, month 2 = 0.88, month 3 = 0.93, month 4 = 0.87) between sunny and shady environments was greater than that of the internal temperature data (month 1 = 0.64, month 2 = 0.61, month 3 = 0.58, month 4 = 0.61). The lowest similarity was found with the radiation values (month 1 = 0.32, month 2 = 0.25, month 3 = 0.24, month 4 = 0.06) (Fig. 1a). The similarity between outdoor and indoor temperature data was greater in the shaded (month 1 = 0.78, month 2 = 0.83, month 3 = 0.85, month 4 = 0.89) than in the sunny (month 1 = 0.44, month 2 = 0.44, month 3 = 0.47, month 4 = 0.43) environment (Fig. 1b).

The highest and lowest mean external and internal temperatures of leaf-cutting *A. sexdens* ant nests in sunny environments over the first 4 months were 24.6, 22.9 °C and 26.2, 24.8 °C respectively and the highest and lowest values of solar irradiance were 21.3 and 17.5 MJ /m^2^ respectively. In the shaded environment, the highest and lowest average external and internal temperatures were 23.5, 22.0 °C and 24.4, 23.3 °C, and the highest and lowest values of solar irradiance were 21.3 and 17.5 MJ/m^2^ respectively (Table 1).

**Table 1:** Average temperature and irradiance during the first (I), second (II), third (III) and fourth (IV) months of *Atta sexdens* (Hymenoptera: Formicidae) nests in sunny and shaded environments.

	I	II	III	IV
**Sunny**				
Internal temperature (°C)	25.3 ± ^1.0^	26.2 ± ^1.0^	26.0 ± ^0.9^	24.8 ± ^1.0^
External temperature (°C)	23.3 ± ^5.8^	24.6 ± ^4.7^	23.9 ± ^4.0^	22.9 ± ^4.1^
Solar irradiation (MJ/m^2^)	21.3 ± ^7.0^	19.0 ± ^8.0^	17.5 ± ^7.1^	17.8 ± ^4.8^
**Shaded**				
Internal temperature (°C)	23.5 ± ^1.8^	24.4 ± ^1.7^	24.3 ± ^1.8^	23.3 ± ^2.1^
External temperature (°C)	22.1 ± ^3.6^	23.5 ± ^3.1^	23.1 ± ^2.8^	22.0 ± ^2.6^
Solar irradiation (MJ/m^2^)	6.3 ± ^3.8^	7.5 ± ^2.0^	3.2 ± ^2.2^	2.3 ± ^1.5^

Superscripted values represent standard deviation.

The ANOVA results indicate no significant effect of the collection date variable or the interaction between the collection dates and the environments. The depth, width, length, height and volume of the initial chamber were greater in *A. sexdens* nests founded in shaded than in sunny environments (Table 2).

**Table 2:** Depth, width, length, height and volume of the initial chamber of *Atta sexdens* (Hymenoptera: Formicidae) nests in sunny and shaded environments.

	Depth*	Width*	Length*	Height*	Volume**
Sunny	9.21 ± 0.39b	2.43 ± 0.14b	3.34 ± 0.18b	2.62 ± 0.15b	8.51 ± 0.12b
Shaded	13.18 ± 0.38a	3.41 ± 0.14ª	4.52 ± 0.16a	3.66 ± 0.14a	24.88 ± 0.15a

Multiple comparison test results.

*Averages followed by the same letter, per column, do not differ by Tukey's test (α = 0.05).

**Medians followed by the same letter, per column, do not differ by Dunn's test (α = 0.05).

The ant queen mass was greater and the fungus garden biomass lower in the first than in the fourth month, 658.5 mg and 64.5 mg and 229.45 mg 1354.9 mg, respectively (Table S1).

The number of *A. sexdens* eggs was lower in the first (25.0) than in the third (115.5) month in the field (Table S1). The number of larvae was similar throughout the 4 months. The number of pupae and small and medium workers was smaller in the first than in the second, third or fourth months (Table S1).

**Figure 2:**
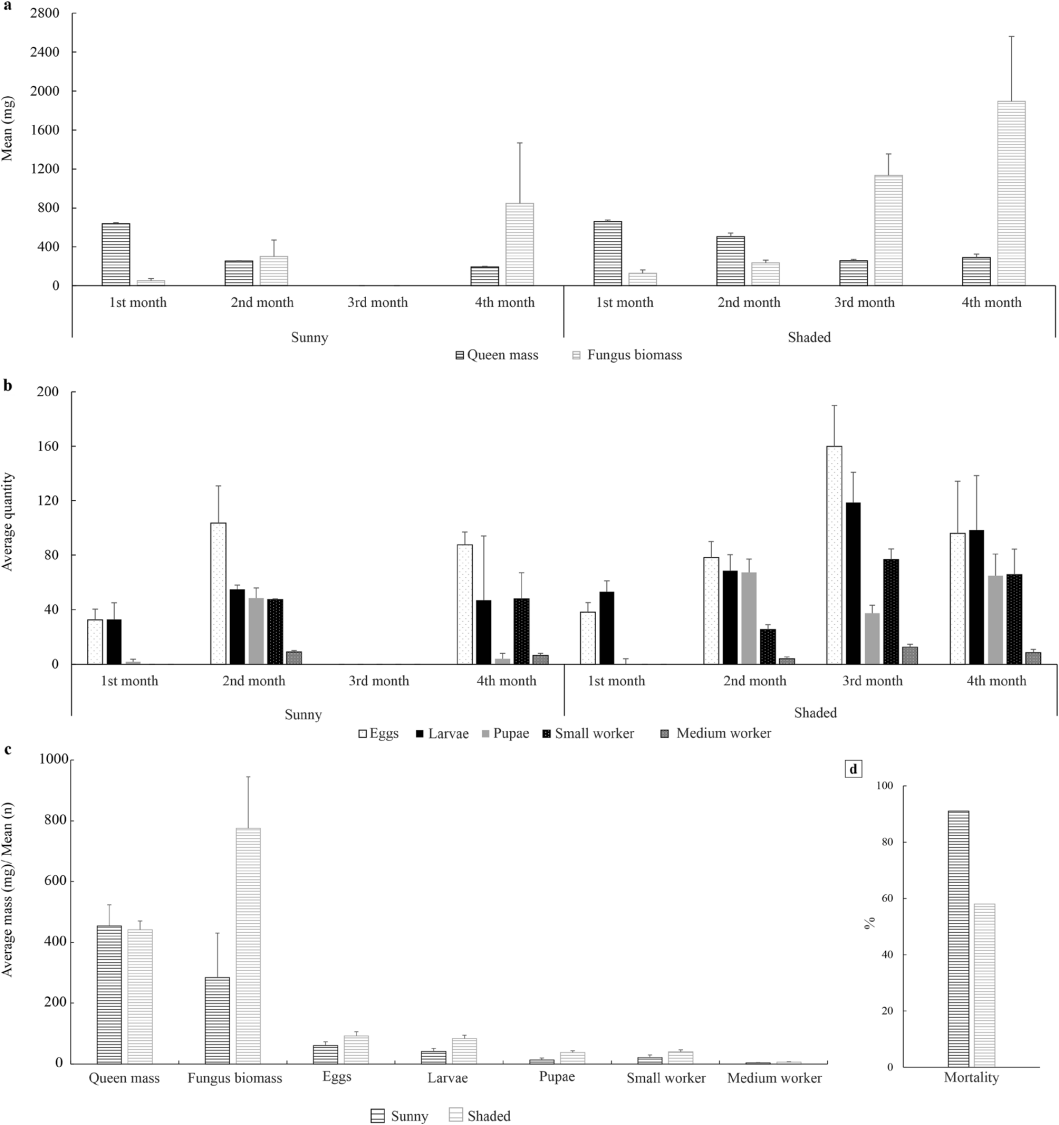
Development of *Atta sexdens* (Hymenoptera: Formicidae) nests in a sunny and shaded environments over 4 months (**a**, **b**) and 4-month average of biology (**c**) and mortality (**d**).

Fungal biomass, queen mass (Fig. 2a) and number of eggs, larvae, pupae and small and medium workers (Fig. 2b) were lower in the sunny than in the shaded environment. The queen body mass was similar between environments (Fig. 2c).

The mortality of *A. sexdens* nests, during the claustral foundation, was higher in the sunny (91%) than in the shaded (58%) (Χ^2^ = 22.79, p < 0.05) environments (Fig. 2d).

## Discussion

The Willmott similarity indices, over the first 4 months, for outside and inside temperatures, in sunny and shaded environments, with values close to 1, confirm that *A. sexdens* colonies were exposed to significantly different temperatures. The lowest values of this index for irradiance, differing between the sunny and shaded environments for internal temperature (in the initial chamber), indicates variation in this parameter between environments. Irradiance values close to 0 between environments represent differences between sunny and shaded areas, but the temperature of the initial chamber did not vary. The selection pressure from irradiance and, consequently, temperature, defines the ideal depth of the initial chamber for the founding queens^16^ to keep this parameter at values adequate for the fungus garden and the offspring^8^ in the field. The similar temperature, in the nests in the shaded environment, agrees with that reported for this leaf-cutting ant, of 24°C^3^, but this varies between species of these insects, being 25–28 °C for *A. sexdens*^17^, 27.5 °C for *A. vollenweideri*^18^ and 27 °C for *A. heyeri*^19^. This adaptation of an ideal depth to construct the initial chamber allows *A. sexdens* to adapt to different habitats in full sun and shade. The nesting process, habits and foraging strategies differ between *A. bisphaerica* and *A. sexdens*, with the former normally establishing their nests in full sun in areas with predominantly grass forages and the latter in shaded areas where it cuts dicot leaves^14^. Furthermore, differences in nest depth between these species may be related to soil temperature, which is lower in shaded areas^7^. For this reason, fungal chambers in exposed nests in pastures are deeper than in shaded areas in forests, as soil temperature is negatively correlated with depth^7^. Soil humidity and temperature act simultaneously due to the thermo preference of workers, resulting in the construction of shallower nests in cold soils and deeper nests in warmer ones^7^. Soil moisture also varies according to depth, affecting the nest-digging behavior of leaf-cutting ants^7,15^.

The temperature in the shaded environment was higher than that reported for *A. sexdens rubropilosa*, from 24.82 ± 3.14 to 24.11 ± 1.30 °C at a depth of 5–25 cm underground, for optimal offspring development and, consequently, reduction in the lipid content of queens at high temperatures, without affecting their survival^20^. This is because the depth of the initial chamber excavated by the queen is adequate for colony success^8,16^.

The different habitats occupied by the ant *A. sexdens*^21^, from dense forests to cerrado and caatinga may explain the greater depth and volume of the initial chamber of the nests in shaded than in sunny environments. However, the depth of the initial nests varies between *Atta* species with 7.5–12 cm, 6.5–13 cm, 15–25 cm, 10–30 cm, 10–15 cm, 11–34 cm, and 9–15 cm for *A. colombica*, *A. cephalotes*, *A. texana*, *A. sexdens rubropilosa*, *A bisphaerica*, *A capiguara,* and *A. insulares*, respectively^1,13,15,22–24^. The initial chamber volume is within the expected range for *A. sexdens*^1,13^ in both environments, with a chamber volume of 24.88 cm^3^ in a shaded area with eucalyptus plantation^13^. Different excavation efforts with the removal of small soil particles by the founding queen using her jaws in repeated biting motions^25^, subsequently discarded outside the nest^8,16,26^ may explain the greater volume of the initial chamber in the shaded area. The greater solar irradiation in sunny areas increases the temperature, with the higher soil temperature generating greater excavation effort and oxidative damage^27,28^, in addition to water loss, as described for seed-collecting ants^29,30^. Further, humid soils are easier to dig, which explains the greater volume of the initial chamber in the shaded area as found for the excavation behavior by *Atta* spp. in soils with different densities and moistures^15,16,31^.

The higher mass of *A. sexdens* queens in the first month of the claustral phase than in the fourth, in both sunny and shaded environments, stems from a reduction in their body mass, due to the metabolism of the lipid content during the first 6 months following the flight, but with recovery in the subsequent months^1^. The mass loss is due to the use of body reserves by the queens to prepare and maintain the colony in the claustral phase, as stored lipids are important in the evolutionary history of the Attini tribe, from semi-claustral to claustral foundation^32^. The queen, with a claustral foundation, does not feed, remaining enclosed in the nest and rearing her initial offspring by metabolizing her own body reserves^33^, as reported for this ant species^1^. The selection pressure on the evolution of claustral foundation tends to minimize risk during foraging^33^ with a more viable adaptation being the storing of reserves in the body, as observed in our study. The greater biomass of the fungus garden in the fourth month is due to growth, but its values were lower than those reported for 4-month-old *A. sexdens* nests, from 2000 to 3000 mg^1^.

The lower number of *A. sexdens* eggs in the first than in the third month is similar to that observed in laboratory colonies of this ant^8^. The irregularity in the egg production by the queen is due to hormone fluctuations regulated by the endocrine system, and is correlated with the activity cycle of the corpora allata during the 3 or 4 months of colony life^34^. This gland synthesizes the juvenile hormone, which acts in the oviposition of founding queens, as verified for females that underwent alatectomy^34^. This hormone acts in the fat body, initiating the synthesis of vitellogenin (glycolipophosphoproteins, with lipids and carbohydrates in its composition) to be deposited in the oocyte^35^. Thus, the production of offspring depends on body reserves (fatty body lipids and muscle mass protein), as the queen does not feed during the foundation period (claustral foundation). The lower production of small and medium workers in the first month than in the second, third or fourth months, agrees with that reported in *A. sexdens* nests^1^. The similar number of larvae over the 4 months is due to the duration of the larval period of *A. sexdens*, of around 25 days^8^, with new immature individuals produced monthly with overlapping generations, common in social insects. This overlap begins with larvae in the first month of nests and pupae, usually in the second month of ant nests in the laboratory^8^.

The lower values of fungus biomass and number of eggs, larvae, pupae and small and medium workers of *A. sexdens* in nests in the sunny environment may be due to a higher incidence of solar irradiance increasing the variation in the internal temperature of the initial chamber. This agrees with reports that a lower incidence of solar irradiance improved the stability of the internal temperature of the initial chamber, favoring *A. sexdens* with narrow thermal tolerance range as it is a thermally protected underground species^11^. However, frequent heat peaks, with habitat-specific physiological consequences for subterranean ectothermic animals, are common in sunny areas^11^. The queen's body mass, similar between environments, indicates a similar reduction of this parameter between them and their tolerance to temperature variations in this type of foundation. A reduction in the mass of *A. sexdens* queens is expected from the nuptial flight to the end of the claustral phase. The energy expenditure of *A. sexdens* queens, in carbohydrates and body lipids for the nuptial flight and nest excavation, was estimated at 0.58 J^36^ and during the claustral phase, they metabolize body lipids and proteins to survive and form the initial colony^26,37^.

The higher mortality of *A. sexdens* nests in the sunny environment, during the claustral foundation, is due to a higher incidence of solar irradiance, increasing the variation in the internal temperature of the initial chamber and, consequently, the excavation effort and oxidative damage to the founding queens^27,28^, in addition to water losses as reported for seed-collecting ants^29,30^. This mortality may also be related to entomopathogens, unsuccessful symbiotic fungus regurgitation, excavation effort, density and soil moisture^1,9,38,39^.

*Atta sexdens* founder queens were exposed to sunny and shaded environments with greater solar irradiance and, consequently, a greater variation range in the internal temperature of the initial chamber in the first environment. The shaded environment, with lower incidence of solar irradiance and greater stability of the internal temperature of the initial chamber, was more favorable for colony development, as confirmed by the biological parameters and greater survival of *A. sexdens* queens.

### *Atta sexdens* female collection methods- after the nuptial flight

*Atta sexdens* queens were collected at the Experimental Farm Lageado in Botucatu, Brazil in 2019 (22°50′37.3"S 48°25′38.3"W) on sunny days after heavy rains from late October to early November. Two hundred queens were collected using tweezers. They were stored separately in 250 ml pots with 1 cm wet plaster for 60 min prior to use. We had permission to collect *Atta sexdens* queen specimens.

### Experimental areas

The *A. sexdens* queens were individualized in two experimental areas: sunny—an open area exposed to Global Horizontal Irradiation with exclusive coverage of *Paspalum notatum* Flügge grass (N = 100) and shaded – an area exposed to Diffuse Horizontal Irradiation (50% shade screen—1.50 × 50 MT), in a plowed environment (N = 100). The soil is a superficial horizon of oxisols.

The founding *A. sexdens* queens were individualized in the center of a square of land (50 × 50 cm) covered with a transparent bottle measuring 20 cm in diameter by 12 cm in height, delimiting the space to be drilled in the soil by the ant queens per experimental area of 25 m^2^ (Fig. S1).

### Development of early nests during the claustral phase

*Atta sexdens* queens were evaluated over 4 months following nest foundation, to monitor its development. A total of 25% of the successfully established nests were excavated per month by removing the colony with a gardening shovel. The number of eggs, larvae, pupae and adults was counted and the mass of the queen and the biomass of the fungus garden determined. The depth, width, length, and height of each nest were measured with the aid of a caliper. The estimated volume of each fungus chamber was based on a cylinder. A correction factor was used to calculate the volume of the chamber because they are rounded: V = πr^2^ (ch + r0.67), in which ‘r’ is the chamber base radius and ‘ch’ the cylinder height, measured by subtracting the maximum height of the chamber from its radius, ch = h − r^40^. Queen mortality was evaluated during the excavation of their nests.

### Temperature and radiation measurement

The temperatures of the external and internal environments (15 cm deep), in each area, were measured for 4 months, with Data loggers (Testo), after the foundation of the nest by the leaf-cutting ant. Global Horizontal Irradiation (GHI) was measured using an Eppley PSP Pyranometer and Diffuse Horizontal Irradiation by a Kipp & Zonen CM3 Pyranometer (Table 3). Solar measurements were obtained over a five-minute time scale (mean of 60 readings with scanning time every five seconds) in W/m^2^ by a CR3000^41^ model data logger and stored in an ASCII file.

**Table 3:** Instruments used to measure solar irradiance in nests of *Atta sexdens* (Hymenoptera: Formicidae) in sunny and shaded environments.

Particulars	Solar irradiance	
Abbreviation	GHI	DHI
Instrument type	Pyranometer	Pyranometer
Maker	Eppley	Kipp & Zonen
Model	PSP	CM3
Spectral range (nm)	295–2800	305–2800

Fonte: Campbell Scientific^42,43^.

The measurements were submitted to a quality control procedure to verify if their values were in accordance with pre-defined solar irradiance thresholds. This procedure consists of a series of checks on physically possible limits per component measured (Table 4). These checks were carried out according to the process created by the International Commission on Illumination (CIE) discarding erroneous measures to avoid compromising the processes of numerical integration or data processing.

**Table 4:** Physically possible minimum and maximum values for each measurement of solar irradiance.

Solar irradiance	Physically possible values (accepted)^44^	
	Minimum	Maximum
GHI	0 W/m^2^	1.20 IE
DHI	0 W/m^2^	I_E_

Measures accepted as possible were those above 0 W/m^2^ and lower than the maximum stipulated limit, per component, according to the extraterrestrial solar irradiance (I_E_) (Eq. 1). This represents the maximum value reaching the top of the atmosphere, without attenuation by atmospheric elements (clouds, particles, among others). Values measured at the earth's surface are lower than those at the top of the atmosphere. However, the phenomenon of multireflection when scattered clouds near the apparent location of the sun reflect part of the solar irradiance onto the sensor, increase the value measured even higher than the extraterrestrial irradiance over short periods^45^. For this reason, the global irradiance value can be up to 20% higher than that of the extraterrestrial one.

The 1361 of Eq. (1), to calculate the extraterrestrial irradiance, represents the solar constant in W/m^2^^46^, R the relation of the average dimensionless distance between the Earth and the Sun (Eq. 2) and Z the zenithal angle of the Sun (Eq. 3) in degrees^47^.

1 $${\text{I}}_{{\text{E}}} = {1361}\left( {\text{1/R}} \right) \,{{\cos}}\, \left( {\text{Z}} \right)$$

2 $$\begin{aligned} {\text{R}} & = {1} - 0.000{9467}\;{\text{sen}} \left( {\text{F}} \right) - 0.0{1671}\;{\text{cos}}\,\left( {\text{F}} \right) - 0.000{1489}\left( {{\text{2F}}} \right) \\ & \quad - 0.0000{2917}\;{\text{sen}}\left( {{\text{3F}}} \right) - 0.000{3438}\;{\text{cos}}\,\left( {{\text{4F}}} \right) \\ \end{aligned}$$

3 $${\text{Z}} = {\text{sen}} \,\left(\updelta \right)\;{\text{sen}}\left(\upphi \right) + \cos \left(\updelta \right)\;\cos \left(\upphi \right)\;\cos \left(\upomega \right)$$

F, in Eq. (4), is the angular fraction of the date of interest in degrees, δ, at 5, the solar declination in degrees, Φ, at 6, the geographic latitude of the location in degrees (22.85) and ω, at 6, the clockwise angle in degrees.

4 $${\mathbf{F}} = {36}0^\circ \;{\text{D/365}}$$

5 $$\begin{aligned} {{\varvec{\updelta}}} & = 0.{3964} + {3}.{631}\;{\text{sen}}\left( {\text{F}} \right) - {22}.{97}\;{\text{cos}}\left( {\text{F}} \right) + 0.0{3838}\;{\text{sen}}\left( {{\text{2F}}} \right) - 0.{3885}\;{\text{cos}}\;\left( {{\text{2F}}} \right) \\ & \quad + 0.0{7659}\;{\text{sen}}\;\left( {{\text{3F}}} \right) - 0.{1587}\;{\text{cos}}\left( {{\text{3F}}} \right) - 0.0{1}0{21}\;{\text{cos}}\left( {{\text{4F}}} \right) \\ \end{aligned}$$

6 $${{\varvec{\upomega}}} = \left( {{12} - {\text{Hd}}} \right){15}$$

The d, in the previous expression, represents the day of the year, from 1 to 365 and the Hd, the hour and the tenth of an hour in degrees of the moment of interest.

The values were numerically integrated, after applying the measurement quality control procedure, obtaining a solar irradiation value for the day in MJ/m^2^ representing the total energy received daily, on a horizontal surface of 1 m^2^.

### Statistical analysis

The null hypothesis that the mortality proportions (probabilities of success) of the founding queens of both groups are the same was submitted to the test of equal proportions.

The null hypothesis that a data sample came from a normally distributed population was submitted to the Shapiro–Wilk test.

Data structure fitting the ANOVA (completely randomized factorial scheme) assumptions were submitted to this analysis and to Tukey's test for multiple comparisons of means. The Scheirer Ray Hare test is a nonparametric test used for a two-way completely randomized factorial design^49^. This procedure is an extension of the Kruskal–Wallis rank test allowing for calculation of the interaction effects and linear contrasts and were used for data structure that did not fit the ANOVA assumptions. Dunn's test^50^ of multiple median comparisons was performed with a correction (the false discovery rate method) to control the experiment-wise error rate.

The Willmott's Index of Similarity (d) is a standardized measure of the degree of similarity between two data series ranging from 0.0 to 1.0 with the value 1.0 indicating a perfect match (two identical data sets), and 0 no agreement at all^51^. As an example, in the sunny environment, the indoor and outdoor temperature data were identical, which results in 1.0. The more identical, close, and concordant two data sets are, the closer to 1.0 they will be. The calculation of the index is presented with A and B representing two data sets whose agreement is to be evaluated.

$$\begin{aligned} A^{\prime}_{i} & = A_{i} - \overline{B} \\ B^{\prime}_{i} & = B_{i} - \overline{B} \\ d & = 1 - \frac{{\sum\nolimits_{i = 1}^{N} {\left( {A_{i} - B_{i} } \right)^{2} } }}{{\sum\nolimits_{i = 1}^{N} {\left[ {\left| {A_{i} } \right| - \left| {B_{i} } \right|} \right]^{2} } }} \\ \end{aligned}$$

The R companion package, ggplot2^52^, FSA^53^, tidyverse^54^, and hydroGOF^55^ used is a free software environment for statistical computing and graphics R version 4.0.4^56^.

## Supplementary Information

Supplementary Information.
