# Supplementary material for: The ideal habitat for leaf-cutting ant queens to build their nests
Source: Sci Rep. 2022 Mar 22;12:4830. doi: 10.1038/s41598-022-08918-2 (PMC8941024; doi:10.1038/s41598-022-08918-2)
Supplement: Supplementary file 1 — Supplementary Information. [file 41598_2022_8918_MOESM1_ESM.doc]

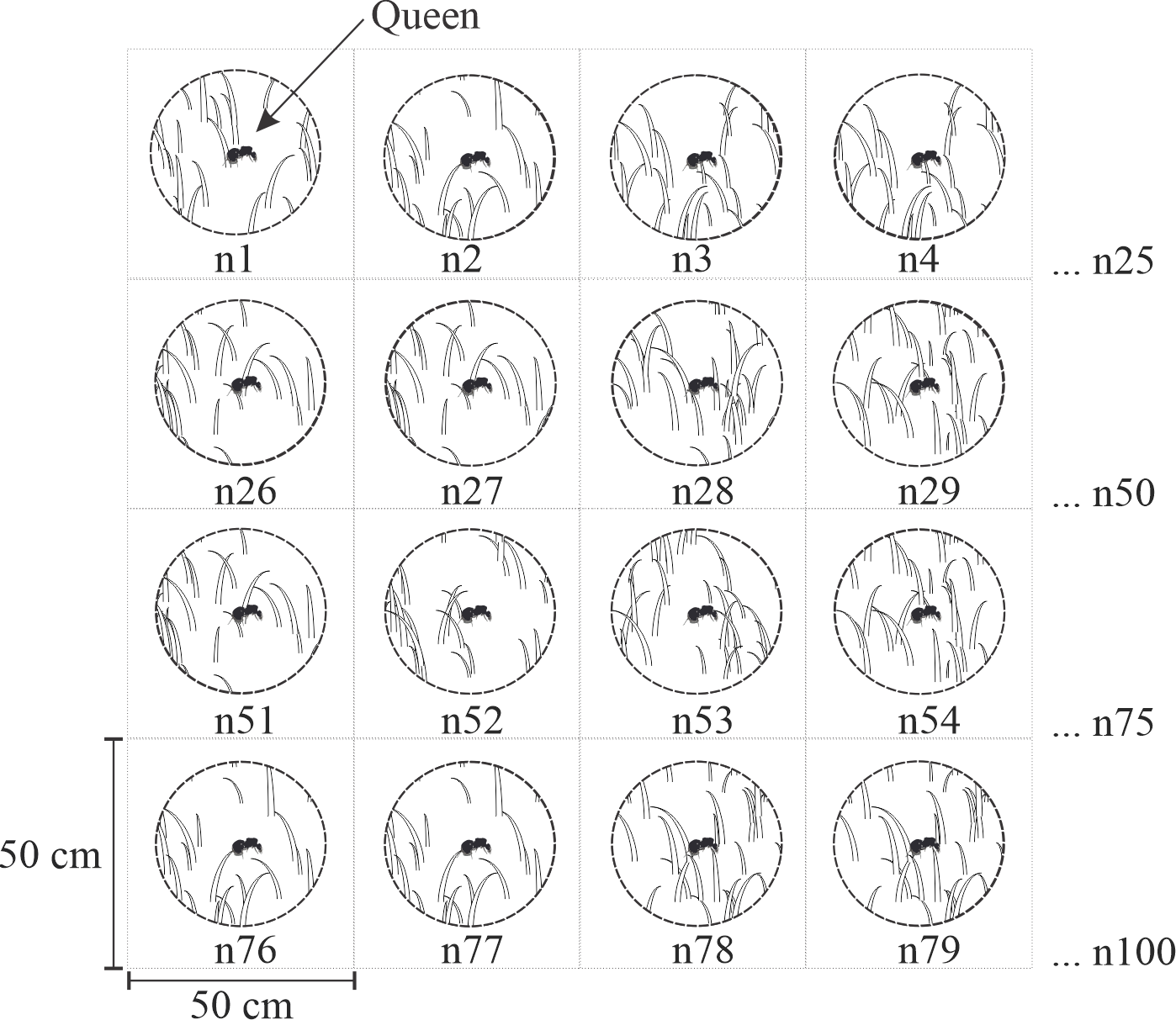
**Figure S1.** Experimental design of the distribution of founder queens of *Atta sexdens* (Hymenoptera: Formicidae) in sunny and shaded environments separated by 800m.

**Table S1.** Queen mass (MR) and fungus biomass (Bio.) (mg), number of eggs (Ov.), larvae (La.), pupae (Pu.) and small (OP) and medium (OM) workers in nests of *Atta sexdens* (Hymenoptera: Formicidae) in the first (I), second (II), third (III) and fourth (IV) months in sunny and shaded environments

|  | | I | | II | | III | | IV | | Sunny | | Shaded |
| --- | --- | --- | --- | --- | --- | --- | --- | --- | --- | --- | --- | --- |
| MR (mg) | 658.5^11.0^a | | 470.7^40.1^b | | 278.3^12.5^c | | 229.4^31.3^c | | 612.8^69.3^a | | 425.45^29.0^a | |
| Bio. (mg) | 64.5^25.4^b | | 189.8^30.7^ab | | 668.3^210.6^a | | 1354.9^523.1^a | | 103.1^146.6^b | | 257.70^170.1^a | |
| Ov. | 25.0^5.3^ª | | 71.5^10.2^b | | 115.5^28.7^b | | 73.5^28.8^ab | | 53.0^12.2^b | | 64.00^13.4^a | |
| La. | 30.0^6.8^a | | 54.0^9.0^a | | 86.5^21.4^a | | 69.0^31.5^a | | 51.0^10.1^b | | 65.50^10.8^a | |
| Pu. | 0.0^2.8^a | | 54.5^8.0^b | | 32.5^5.6^b | | 62.5^14.2^b | | 0.0^6.6^b | | 32.50^5.7^a | |
| OP. | 0.0^0^a | | 25.0^3.8^b | | 64.0^7.4^c | | 66.0^14.5^bc | | 0.0^8.5^b | | 28.50^6.4^a | |
| OM. | 0.0^0^a | | 3.0^1.2^b | | 8.5.0^2.0^b | | 7.5^1.8^b | | 0.0^1.3^b | | 4.50^1.1^a | |

Multiple Comparison Test Results. *Medians followed by the same letter, per line, do not differ by Dunn's test (α= 0.05). Superscript values are standard errors.
